# Membranous nephropathy associated with thrombospondin type-1 domain-containing 7A (THSD7A) in an adult woman with eosinophilia

**DOI:** 10.1007/s13730-019-00430-3

**Published:** 2019-11-08

**Authors:** Sayuri Shirai, Shin’ichi Akiyama, Atsuko Kamijo-Ikemori, Tomo Suzuki, Daisuke Ichikawa, Junki Koike, Kenjiro Kimura, Yugo Shibagaki

**Affiliations:** 1 Division of Nephrology and Hypertension, Department of Internal Medicine, St. Marianna University School of Medicine Yokohama City Seibu Hospital, 1197-1 Yasashi-cho, Asahi-ku, Yokohama, 241-0811 Japan; 2 Division of Nephrology, Department of Internal Medicine, Nagoya University Graduate School of Medicine, 65 Tsurumai-cho, Showa-ku, Nagoya, 466-8550 Japan; 3 Department of Anatomy, St. Marianna University School of Medicine, 2-16-1 Sugao, Miyamae-ku, Kawasaki, 216-8511 Japan; 4 Department of Nephrology, Kameda Medical Center, 929 Higashicho, Kamogawa, 296-8602 Japan; 5 Division of Nephrology and Hypertension, Department of Internal Medicine, St. Marianna University School of Medicine, 2-16-1 Sugao, Miyamae-ku, Kawasaki, 216-8511 Japan; 6 Department of Diagnostic Pathology, St. Marianna University School of Medicine, 2-16-1 Sugao, Miyamae-ku, Kawasaki, 216-8511 Japan; 7 Division of Nephrology, Department of Internal Medicine, Tokyo Takanawa Hospital, 3-10-11 Takanawa, Minato-ku, Tokyo, 108-8606 Japan

**Keywords:** Membranous nephropathy, Eosinophilia, THSD7A, PLA2R

## Abstract

A 30-year-old woman on steroid therapy for eosinophilia presented with nephrotic syndrome during steroid tapering. She was diagnosed with membranous nephropathy (MN) stage II–III (positive for IgG1 and IgG4) by renal biopsy. There was no evidence of secondary MN. Her urinary protein level was controlled to 0.5 g/day or less, and her eosinophil count in white blood cell differential was stabilized at less than 10% without increasing the steroid dosage. The renal specimen did not show any enhanced granular expression of PLA2R along the glomerular basement membrane, and PLA2R was not detected in the patient’s serum. On retrospective analysis, enhanced granular staining for thrombospondin type-1 domain-containing 7A (THSD7A) in the glomeruli was detected in the biopsy, and anti-THSD7A IgG was detected in the serum using a commercial indirect immunofluorescence test (IFT). Based on these, the case was considered as THSD7A-associated MN with comorbid eosinophilia. The causal relationship between THSD7A-related MN and eosinophilia was unclear. However, a few cases of THSD7A-associated MN with eosinophilia have been reported, and further clarification on the relationship between THSD7A-related MN and eosinophilia is warranted.

## Introduction

Membranous nephropathy (MN), a common nephrotic syndrome in adults, is caused by immune complex deposition in the subepithelial area of the glomerular basement membrane and the subsequent activation of the complement system. About 20% of MNs are secondary MNs that present along with malignant tumors, drug reactions, collagen diseases, and infectious diseases; and most of them are idiopathic.

Recently, it was reported that M-type phospholipase A2 receptor 1 (PLA2R) and thrombospondin type-1 domain-containing 7A (THSD7A) are two of the causative antigens of idiopathic MN (IMN) [1, 2]. The prevalence of PLA2R-associated MN and THSD7A-associated MN were reported to be 80–85% and 3–5%, respectively [3]; in Japan, they are 50–70%, and 3–10%, respectively [4]. Iwakura et al. reported that 60% of women with THSD7A-associated MN were diagnosed before the age of 40 years [5].

Recent studies have also shown a possible relationship between THSD7A-associated MN and cancer [6, 7]. Hoxha et al. screened serum samples of 1276 patients with MN from three different cohorts for the presence of THSD7A-Ab using a newly developed indirect immunofluorescence test (IFT) and showed that the new test had 92% sensitivity and 100% specificity. Twenty percentage of the patients with THSD7A-associated MN in their cohort also had a malignant disease, while only 7% with PLA2R1-associated MN presented with cancer [6]. However, other causes of THSD7A-associated MN remains unknown. Recently, there have been a few reports of THSD7A-associated MN that were complicated by allergic diseases [8–10]. The credibility of this association requires assessment; however, we have experienced similar cases firsthand.

Here, we report a case of THSD7A-associated MN with comorbid eosinophilia. THSD7A was detected in the glomeruli by immunohistochemistry on frozen kidney biopsy samples. In addition, anti-THSD7A IgG in the serum was detected using a commercial indirect IFT. Our findings indicate the necessity to examine the relationship between allergic diseases and THSD7A antibody generation.

## Case report

A 30-year-old woman, in March X-1 year, after wisdom tooth extraction, manifested bilateral arthralgia, swelling of lower legs and forearms, and pain on Achilles tendon sticking area. She visited a primary care clinic, where her blood test showed an increase in WBC count and was referred to a city hospital. Her WBC count was 20,710/µL; eosinophils 10,562/µL (51%). Urine examination reported protein, 1+, and occult blood, 1+. WBC increase had not been detected during screening. She had a history of pediatric asthma and allergic rhinitis. She had no prior infection and no history of oral supplements. She was negative for C-reactive protein (CRP), rheumatoid factor (RF), antinuclear antibody (ANA), and antineutrophil cytoplasmic antibody (ANCA). Her chest X-ray was normal. Based on these observations, she was diagnosed with eosinophilia and was transferred to the rheumatology clinic in our institute at the end of March X-1 year. At that time, her blood and urine examinations showed the following: WBC, 15,400/µL; eosinophils, 4774/µL (31%); serum IgE, 530 IU/mL; urine protein, 3+; and urine occult blood, 1+. The patient was administered 15-mg oral prednisolone (PSL) for eosinophilia; the dose was gradually reduced. After PSL administration, her blood and urine examination showed remission of proteinuria and normal eosinophil count; however, after decreasing PSL dose to 4 mg, her urinary protein increased to over 1 g/g Cr.

She underwent emergency surgery for appendicitis in December X-1 year (at that time, the PSL dosage was 4 mg). About a month after the surgery, she became aware of edema in the lower limbs, and by February X year her weight increased by 3–5 kg. Her proteinuria increased to 8.9 g/g Cr in March 2010.

**Fig. 1:**
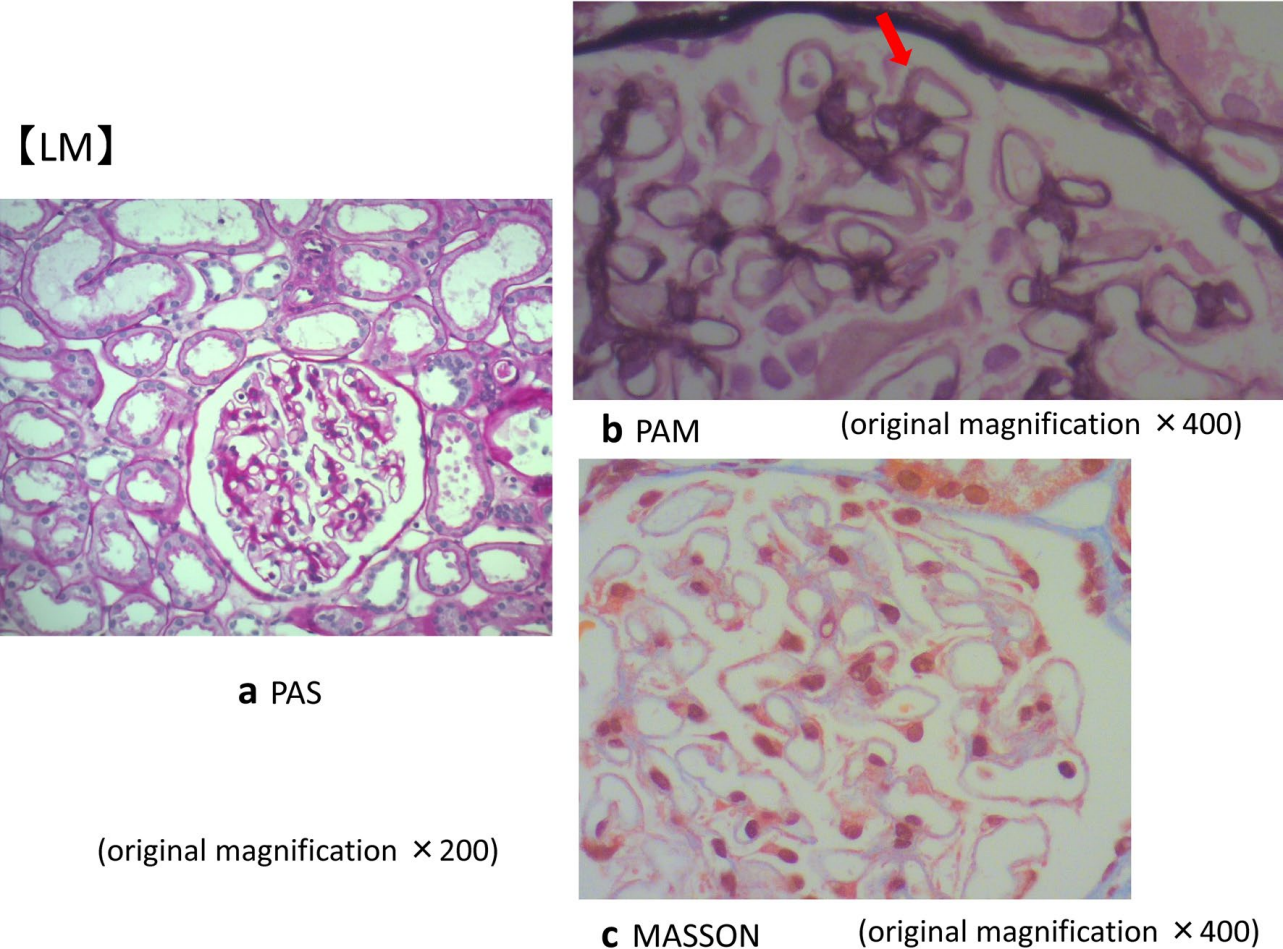
Micrographs showing glomeruli in the kidney. **a** Periodic acid-Schiff (PAS) staining of the renal biopsy specimen showing a minor glomerular abnormality. Original magnification × 200. **b** Periodic acid methenamine silver (PAM) staining showing focal segmental spike formations (red arrow). Original magnification × 400. **c** Masson trichrome staining showing granular immune complex along the glomerular basement membrane. Original magnification × 400

**Fig. 2:**
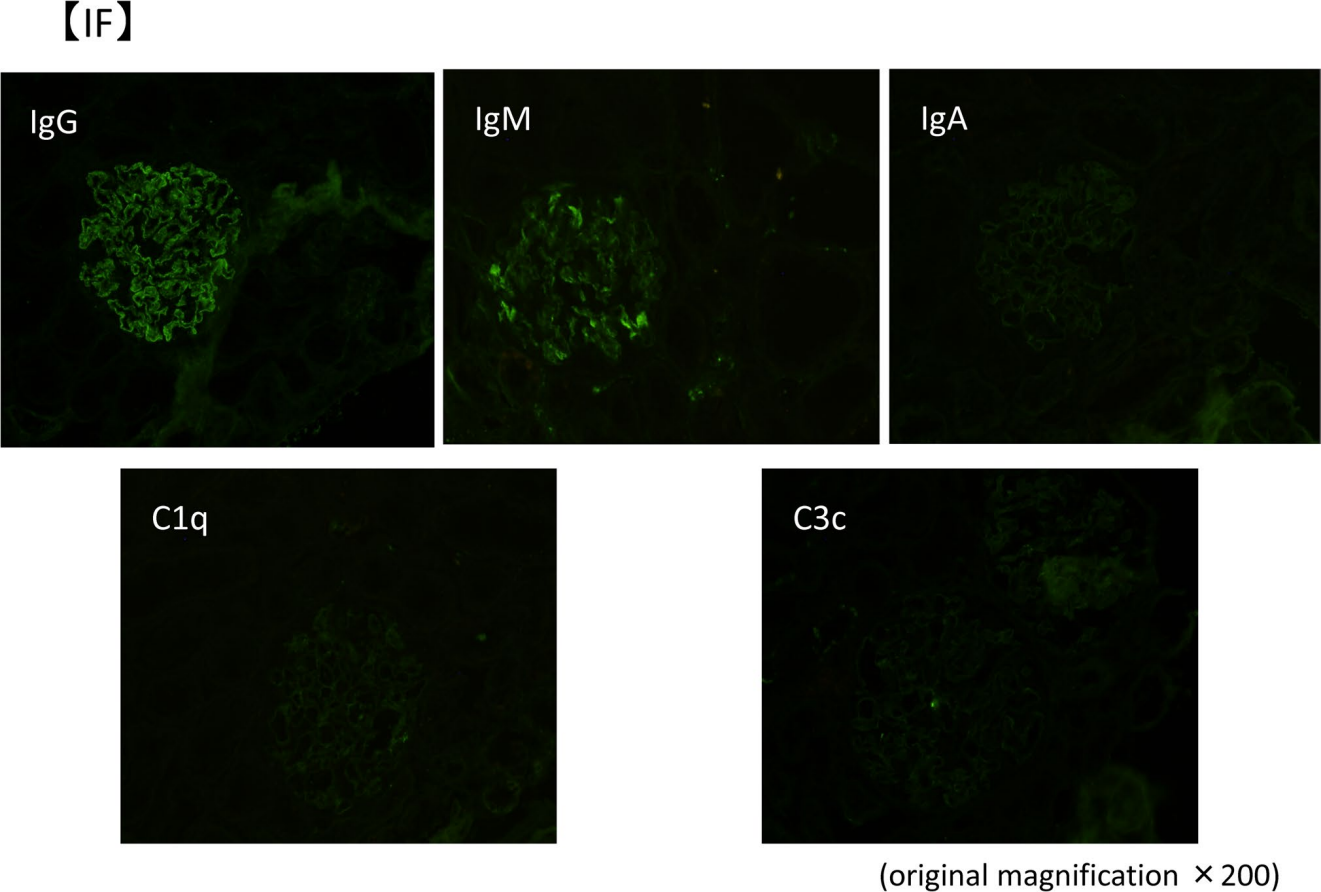
Immunofluorescence microscopy (IF). IF image of the renal specimen showing granular 2 + capillary wall staining for IgG, 1 + capillary wall staining for IgM. No staining for IgA, C1q, C3c. Original magnification × 200

**Fig. 3:**
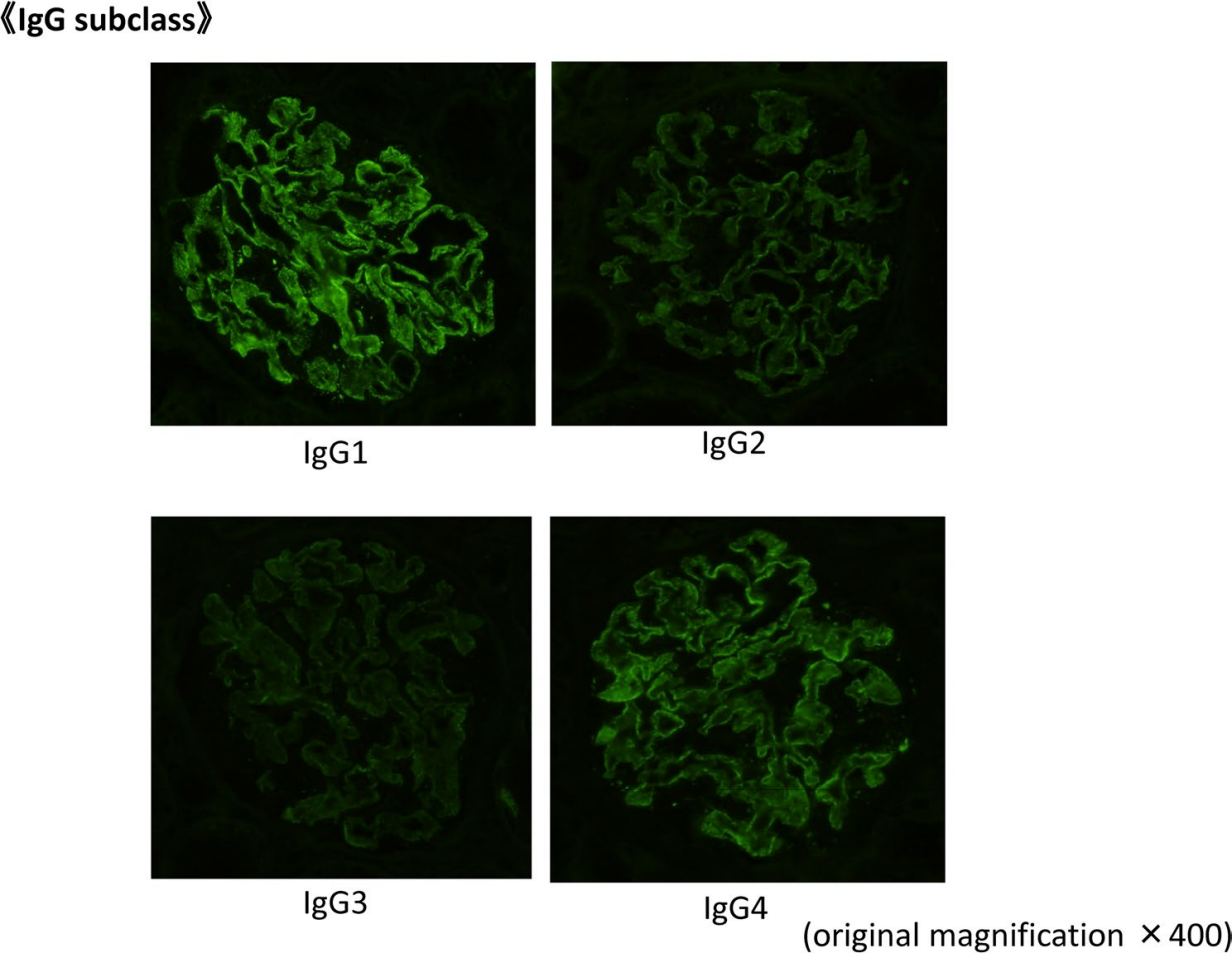
IgG subclass staining in IF IgG1 and IgG4 IF image of the renal specimen showing granular 2 + capillary wall staining for IgG1 and IgG4. No staining was observed for IgG2 and IgG3. Original magnification × 200

**Fig. 4:**
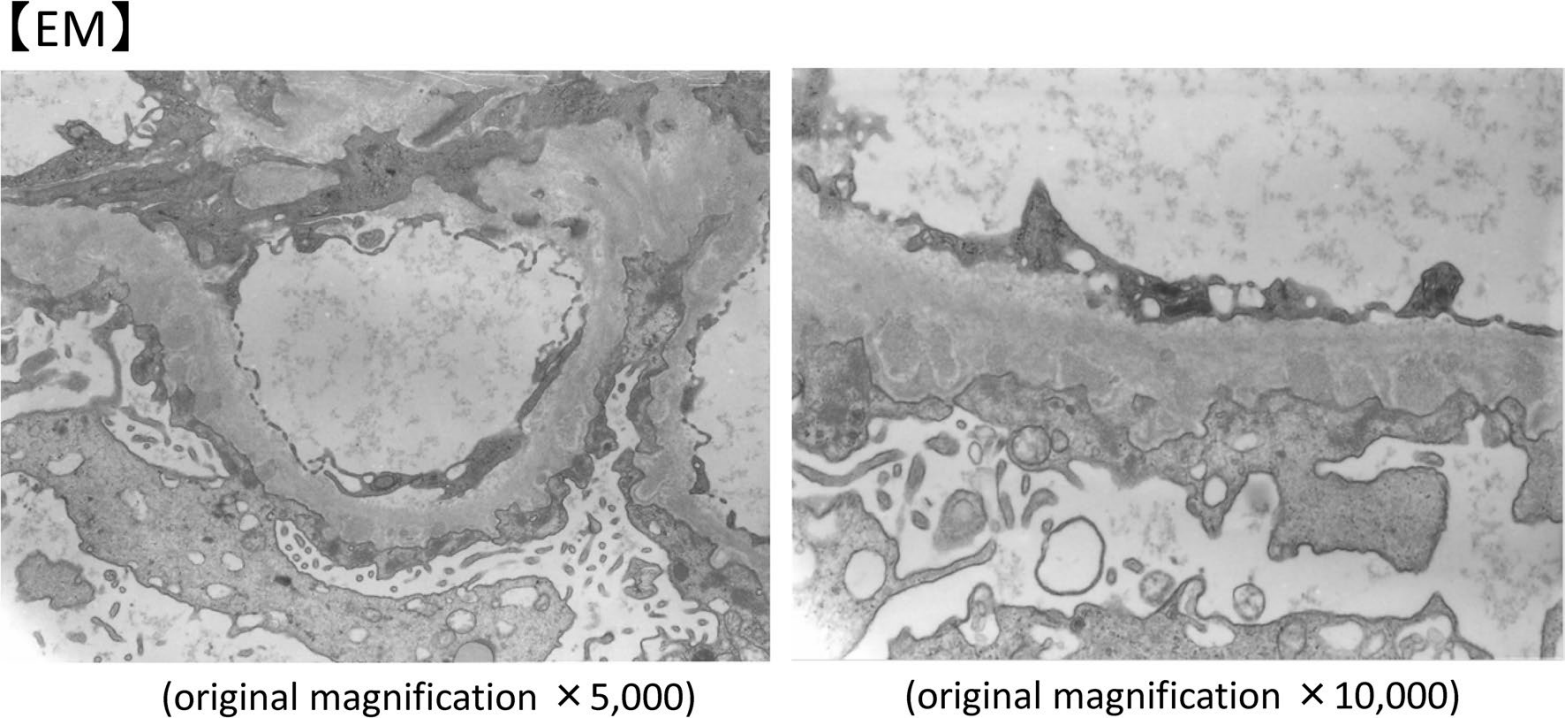
Electron microscopy (EM). EM image of the renal specimen revealed subepithelial deposits in the glomerular basement membrane. Original magnification × 5000 and × 10,000

A kidney biopsy was performed in May X year. Table 1 shows the laboratory data at the time of renal biopsy. At that time, eosinophils were suppressed by the steroid treatment. There were focal segmental spike formations in Periodic acid methenamine (PAM) staining (Fig. 1b). Granular IgG deposits along the capillary basement membrane (Fig. 2) were identified by immunofluorescence microscopy (IF). Codominant staining of IgG1 and IgG4 (Fig. 3) was observed. Electron microscopy classified the subepithelial deposits as stage II ~ III (Fig. 4).

**Table 1:** Laboratory data of the patient (under steroid treatment) at the time of renal biopsy

Complete blood count		Serology	
WBC	9.9×10^3^/μL	C3	88 mg/dL
Eosinophils	5.6%	C4	17 mg/dL
Hb	13.1 g/dL	IgG	540 mg/dL
Plt	40.9×10^4^/μL	IgA	106 mg/dL
Blood chemistry		IgM	249 mg/dL
T-bil	1.0 mg/dL	IgE−RIST	330 IU/ml
AST	12 IU/L	RF	(−)
ALT	10 IU/L	ANA	(−)
ALP	144 IU/L	MPO−ANCA	< 10 EU
LDH	169 IU/L	PR3−ANCA	< 10 EU
TP	4.6 g/dL	Urine analysis	
Alb	2.8 g/dL	Protein	3.2 g/gCr
BUN	7.7 mg/dL	NAG	13.5 U/L
Cr	0.49 mg/dL	RBC	1–4/HPF
eGFR	117.9 mL/min/1.73m^2^	WBC	10–19/HPF
UA	7.4 mg/dL		
Na	139 mEq/L		
K	4.2 mEq/L		
Cl	111 mEq/L		
CRP	< 0.03 mg/dL		
HbA1C	4.6%		

*RBC* red blood cell count, *WBC* white blood cell count, *Hb* hemoglobin, *Plt* platelet, *T-bil* total bilirubin, *AST* aspartate aminotransferase, *ALT* alanine aminotransferase, *ALP* alkaline phosphatase, *LDH* lactic dehydrogenase, *TP* total protein, *Alb* albumin, *BUN* blood urea nitrogen, *Cr* creatinine, *UA* uric acid, *CRP* C-reactive protein, *C3* complement component 3, *C4* complement component 4, *Ig* immunoglobulin, *RF* rheumatoid factor, *ANA* antinuclear antibodies, *ANCA* antineutrophil cytoplasmic antibody, *MPO-* myeloperoxidase-, *PR3-* protease 3-, *NAG* N-acetyl-β-D-glucosamidase

**Fig. 5:**
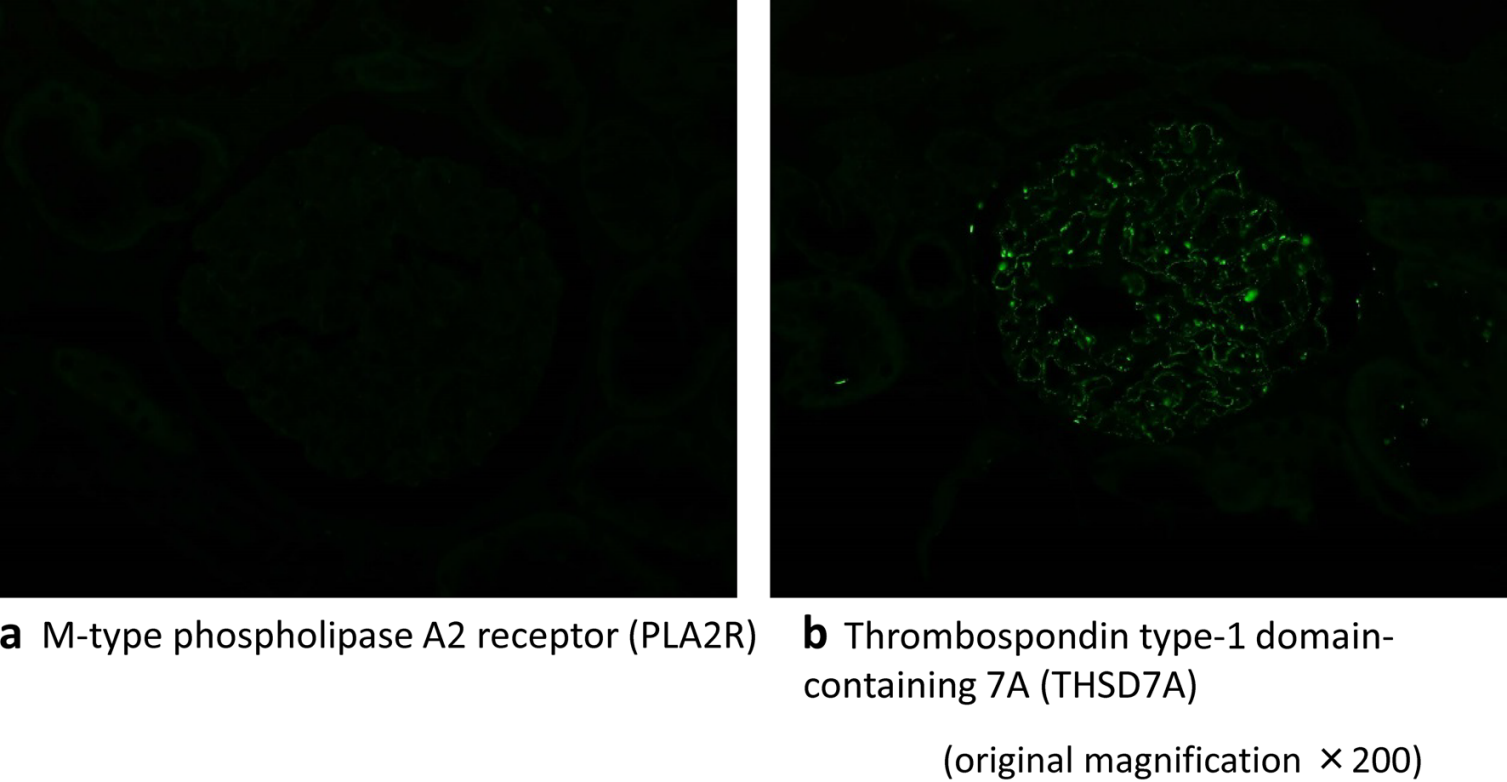
Immunofluorescence staining in the glomeruli. **a** Immunofluorescence imaging of a renal specimen from the patient did not show any enhanced granular expression of M-type phospholipase A2 receptor (PLA2R) along the glomerular basement membrane. **b** Immunofluorescence image of renal specimen from the patient showing granular staining for thrombospondin type-1 domain-containing 7 A (THSD 7 A) along the glomerular basement membrane. Original magnification × 200

**Fig. 6:**
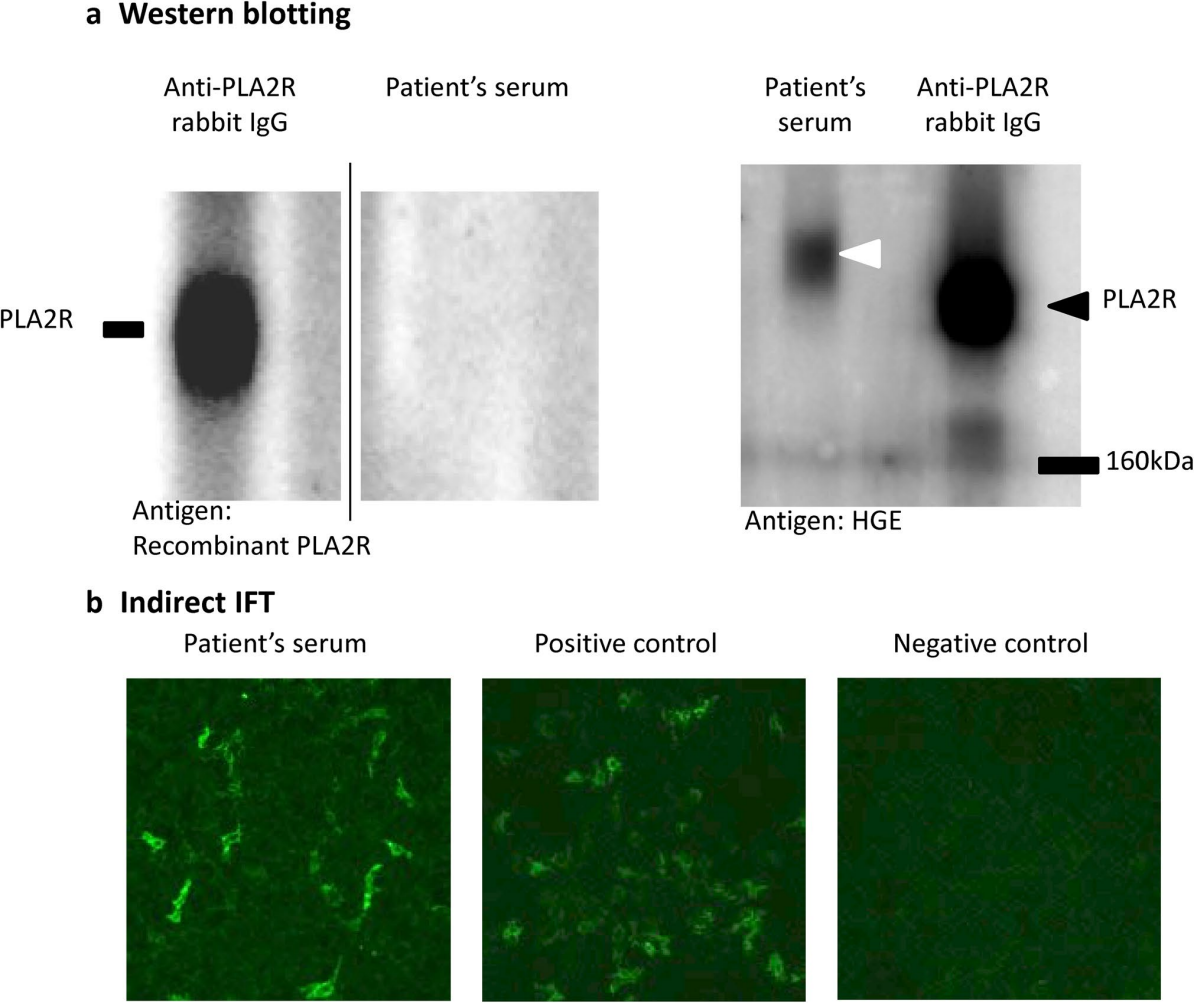
Detection of circulating anti-THSD7A IgG. Anti-THSD7A antibody was measured by western blot analysis and commercial indirect immunofluorescence test (IFT). Western blot analyses of human glomerular extract (HGE) and recombinant PLA2R protein were performed under non-reducing conditions with patient’s serum, using a commercial anti-PLA2R rabbit polyclonal antibody as the primary antibody. The bands were detected by chemical luminescence imaging with HRP-labeled anti-human IgG mouse monoclonal antibody and HRP-labeled anti-rabbit IgG goat polyclonal antibody. A commercial indirect IFT for anti-THSD7A IgG (Euroimmun) was performed with the patient’s serum according to the manufacturer’s protocol. **a** The image on the left shows the result of western blotting of recombinant PLA2R, expressed in HEK293 cells, under non-reducing condition with patient’s serum and anti-PLA2R rabbit polyclonal antibody. The patient’s serum did not recognize the recombinant PLA2R protein. The image on the right shows the result of western blotting of the HGE under non-reducing condition with patient’s serum and anti-PLA2R rabbit polyclonal antibody. The patient’s serum recognized a single band (white arrowhead) whose molecular weight was higher than that of PLA2R (black arrowhead) which was subsequently identified as THSD7A. **b** Fluorescence microscopy images obtained using a commercial indirect IFT. The patient’s serum bound to the recombinant THSD7A protein on the cell surface

Next, we used an anti-PLA2R antibody to screen for the PLA2R antigen responsible for MN. Immunofluorescence imaging of a renal specimen did not show enhanced granular expression of PLA2R along the glomerular basement membrane (Fig. 5a). PLA2R was not detected in the patient’s serum by western blotting (Fig. 6a left). Based on the above results, we initially diagnosed the condition as IMN However, a protein with a molecular weight higher than that of PLA2R was detected by western blot (Fig. 6a right).

**Fig. 7:**
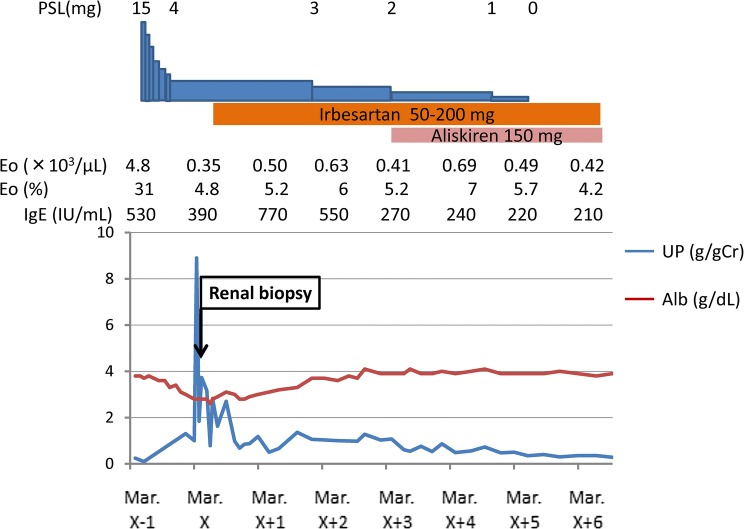
Clinical course and treatment of the patient. *Eo* eosinophil, *IgE* Immunoglobulin E, *UP* urine protein, *Alb* albumin, *PSL* prednisolone

As the proteinuria spontaneously improved, PSL dosage was maintained at 4 mg. An incomplete remission (urine protein < 1 g/g Cr) was attained in about half a year. From October X year, PSL dosage was reduced to 3 mg. After that, PSL dosage was reduced more gradually; PSL was discontinued in March X + 5 years; the proteinuria dropped to less than 1 g/g Cr. The patient was maintained on irbesartan, and aliskiren and the proteinuria further dropped to 0.5 g/g Cr. The eosinophil count was also stabilized at less than 10% and was maintained at a slightly higher average of 5.6% with PSL 4 mg/day or less. (Fig. 7).

In the meantime, THSD7A was identified as the second antigen responsible for IMN in X + 4 years. We retrospectively identified the heavier band detected by western blotting as THSD7A, which was supported by the following findings: enhanced granular staining for THSD7A in the glomeruli in a biopsy specimen (Fig. 5b), and detection of anti-THSD7A IgG in the serum using a commercial indirect IFT (Fig. 6b). Based on these, we diagnosed this case as THSD7A-associated MN.

## Discussion

Autoantibodies against podocyte antigen, thrombospondin type-1 domain-containing 7A (THSD7A), have been reported to be responsible for primary MN; but, it remains uncertain how these antigens are recognized by the immune system.

In the present case, WBC and eosinophil count increased at the same time as reactive arthritis, so it cannot be denied that reactive arthritis caused the eosinophil increase [11]. There was a history of allergic rhinitis and childhood asthma as well as an underlying allergic predisposition. We speculate that reactive arthritis may have developed due to some kind of trigger after the dental treatment. A renal biopsy was performed 1 year and 2 months after the onset of eosinophilia. There was no eosinophil infiltration in the kidney tissue, probably because the eosinophil count of 1500/μL or more did not last long enough under steroid treatment. The time when the urinary abnormality was observed coincided with the onset of eosinophilia; suggesting that eosinophilia and MN, which were later diagnosed, may have been present since then. Proteinuria in the nephrotic range appeared when the steroid dose was reduced from 15 to 4 mg, and the pre-existing MN may have been exacerbated due to the stress of surgery for appendicitis, as supporting evidence, the stage of MN was 2–3.

In 2014, Tomas et al. reported THSD7A, a type I membrane protein (250 kDa) expressed in the glomerular epithelial cells, as a new endogenous antigen of human MN [2]. An immunofluorescence examination of the renal specimen from our patient showed an enhanced granular expression of THSD7A along the glomerular basement membranes. Additionally, we confirmed THSD7A positivity by western blotting and indirect IFT for anti-THSD7A IgG detection. We have recently recognized this case as THSD7A-associated MN. However, we had recognized a single band whose molecular weight was higher than that of PLA2R in the patient’s serum by western blotting before Tomas et al.’s report [2] was published.

There are several reports of MN complicated with eosinophilia [12–14]. Although the relationship between eosinophilia and MN is not clear, several immunological mechanisms are conceivable. It has been reported that the immunological background of MN patients has superiority to Th2 [15] and that Th2 cytokines induce IgG4 synthesis [16]. Meanwhile, it is known that Th2 cytokines are produced by natural killer cells, eosinophils, and mast cells, in addition to Th2 cells. It has been speculated that viral infections or toxins may stimulate the release of lymphokines by altering T cell immunoregulation. These immunological triggers could result in associated renal lesions [13, 17]. Regarding the relationship between eosinophilia and THSD7A-associated MN, Matsumoto et al. presented two cases of THSD7A-associated MN accompanied by ALHE, a benign tumor characterized by the proliferation of plump endothelial cells [8]. They found that in ALHE, eosinophils expressed vascular endothelial growth factor-A (VEGF-A), which upregulated THSD7A expression, especially under Th2-prone conditions in cultured human umbilical vein endothelial cells (HUVEC). Their results showed that VEGF-A-induced THSD7A expression outside the kidney might be necessary for MN pathogenesis. Furthermore, Suzuki et al. reported a case of THSD7A-MN with refractory asthma and eosinophilia and suggested that THSD7A-MN may be associated with severe asthma and eosinophilia [9]. In this case, THSD7A-associated MN is thought to have occurred coincidentally with eosinophilia, but it cannot be completely denied that eosinophilia caused THSD7A-associated MN. It could be considered that eosinophilia caused some endothelial cell injury, which induced THSD7A, as stated in previous reports.[8, 9]. Recently, Hara et al. reported that among 469 consecutive cases of pathologically confirmed MN diagnosed by THSD7A tissue staining of renal biopsy specimens at four centers in Japan, 14 cases were confirmed positive for THSD7A by immunohistochemistry (3.0%), and four patients had concurrent or previous incidence of allergic diseases, including one patient with Kimura’s disease [10].

In summary, we report a case of THSD7A-associated MN in an adult woman with eosinophilia diagnosed by serum antibody testing as well as THSD7A staining in a renal biopsy. Only a few cases of allergic disease such as eosinophilia complicated with THSD-related MN have been reported, and details regarding its relevance are unknown. Further investigation based on a nationwide disease registry is required to understand the relationship between THSD7A-related MN and eosinophilia.
